# Inhibition of homoserine dehydrogenase by formation of a cysteine-NAD covalent complex

**DOI:** 10.1038/s41598-018-24063-1

**Published:** 2018-04-10

**Authors:** Kohei Ogata, Yui Yajima, Sanenori Nakamura, Ryosuke Kaneko, Masaru Goto, Toshihisa Ohshima, Kazuaki Yoshimune

**Affiliations:** 1 Department of Biomolecular Science, Graduate School of Science, Toho University, 2-2-1, Miyama, Funabashi, Chiba 274-8510 Japan; 2 Department of Applied Molecular Chemistry, College of Industrial Technology, Nihon University, 1-2-1, Izumichou, Narashino, Chiba 275-8575 Japan; 3 Department of Applied Molecular Chemistry, Graduate School of Industrial Technology, Nihon University, 1-2-1, Izumichou, Narashino, Chiba 275-8575 Japan; 4 Department of Biomedical Engineering, Osaka Institute of Technology, 5-16-1, Ohmiya, Asahi-ku, Osaka 535-8585 Japan

## Abstract

Homoserine dehydrogenase (EC 1.1.1.3, HSD) is an important regulatory enzyme in the aspartate pathway, which mediates synthesis of methionine, threonine and isoleucine from aspartate. Here, HSD from the hyperthermophilic archaeon *Sulfolobus tokodaii* (StHSD) was found to be inhibited by cysteine, which acted as a competitive inhibitor of homoserine with a *K*i of 11 μM and uncompetitive an inhibitor of NAD and NADP with *K*i’s of 0.55 and 1.2 mM, respectively. Initial velocity and product (NADH) inhibition analyses of homoserine oxidation indicated that StHSD first binds NAD and then homoserine through a sequentially ordered mechanism. This suggests that feedback inhibition of StHSD by cysteine occurs through the formation of an enzyme-NAD-cysteine complex. Structural analysis of StHSD complexed with cysteine and NAD revealed that cysteine situates within the homoserine binding site. The distance between the sulfur atom of cysteine and the C4 atom of the nicotinamide ring was approximately 1.9 Å, close enough to form a covalent bond. The UV absorption-difference spectrum of StHSD with and without cysteine in the presence of NAD, exhibited a peak at 325 nm, which also suggests formation of a covalent bond between cysteine and the nicotinamide ring.

## Introduction

Among the various amino acid metabolic pathways found in plants and most bacterial strains, the aspartate pathway is pivotal because it is responsible for the production of multiple amino acids, including lysine, threonine, methionine, and isoleucine^1^. Within the aspartate pathway to threonine, methionine and isoleucine synthesis, homoserine dehydrogenase (HSD) functions in the third reaction step – i.e., NAD(P)H-dependent production of homoserine from L-aspartate-4-semialdehyde. HSD is known to be susceptible to regulation by various amino acids and other pathway intermediates. Moreover, because the aspartate pathway is essential for plants, fungi, and bacteria, but is absent in mammals, HSD inhibitors are attractive as potential antibiotics and herbicides.

*Escherichia coli* express two types of bifunctional HSDs: one that is inhibited by cysteine, serine, and threonine^2^, and another that is inhibited by methionine^3^. The HSD from *Corynebacterium glutamicum* is inhibited by threonine, and its C-terminus is responsible for its allosteric regulation^4^. However, much less is known about the functional and regulatory mechanisms and structural characteristics of archaeal HSDs than the bacterial enzymes, though the characteristics of HSD from *Pyrococcus horikoshii* (PhHSD) were recently reported^5^. PhHSD utilizes NAD, but not NADP, as an active coenzyme. NADP functions as a strong dead end inhibitor of NAD-dependent activity, and NADPH was found in the coenzyme binding site in the crystal structure^5^. We previously reported that the HSD from the hyperthermophilic archaeon *Sulfolobus tokodaii* (StHSD) is activated by reductive cleavage of the disulfide bond formed between cysteine residues (Cys304) in the C-terminal regions of the homodimer subunits^6^. The crystal structure revealed that StHSD is composed of a nucleotide-binding region (residues 1–130 and 285–304), a dimerization region (residues 131–145 and 256–284), and a catalytic region (residues 146–255)^6^. In the present study, we discovered the marked inhibition of StHSD by cysteine and present structural evidence that a cysteine binds at the active site of StHSD in complex with NAD.

## Results

### Cysteine inhibition and kinetic analysis

Cysteine (10 mM) markedly inhibited NAD-dependent homoserine oxidation by purified StHSD (95% inhibition). The effect exhibited the features of competitive inhibition of homoserine, with a calculated *K*i of 11 μM. In addition, cysteine also exerted an uncompetitive inhibitory effect against NAD, with a calculated *K*i of 0.55 mM. By contrast, StHSD was inhibited by less than 5% by 10 mM methionine, isoleucine, or threonine, all of which are final products in the aspartate pathway. Lysine (10 mM), which is produced via the AAA pathway in *Sulfolobus* species, had little effect on StHSD activity. Weak inhibition (14%) by serine (10 mM), a homoserine analog, was also observed.

To investigate the reaction mechanism, we plotted the reciprocals of the initial velocities against the reciprocals of the NAD concentrations at several different homoserine concentrations, which gave a set of straight lines that intersected in the upper left quadrant. This suggests NAD-dependent homoserine oxidation catalyzed by StHSD proceeds via an ordered mechanism. Therefore product inhibition of NAD-dependent homoserine oxidation was examined using the method of Cleland to determine the order of substrate binding^7^. In the presence of several different concentrations of NADH used as an inhibitor, double reciprocal plots of velocity against NAD concentration at a high constant homoserine concentration (10 mM, about 60x the apparent *K*m value) produced a set of straight lines intersecting at a point on the vertical axis, which suggests that NADH acts as a competitive inhibitor of NAD. In addition, double reciprocal plots of velocity against homoserine concentration at a high constant NAD concentration (10 mM, about 30x the apparent *K*m value) in the presence of several NADH concentrations gave a set of straight lines intersecting at a point in the second quadrant, which suggests non-competitive inhibition by homoserine. The initial velocity pattern and two product inhibition patterns suggest that homoserine oxidation by StHSD proceeds through a sequentially ordered mechanism in which NAD binds to the free form of the enzyme, after which homoserine binds to the enzyme-NAD complex.

StHSD also exhibited activity with NADP. The apparent *V*_max_ and *K*_m_ were 0.38 U/mg and 1.2 mM, respectively, which is much lower than the apparent *V*_max_ and *K*_m_ for NAD (1.3 U/mg and 0.33 mM, respectively), as reported previously^6^. The inhibition of NADP by cysteine was uncompetitive, with a *K*i of 1.2 mM.

### Structure of StHSD

**Figure 1:**
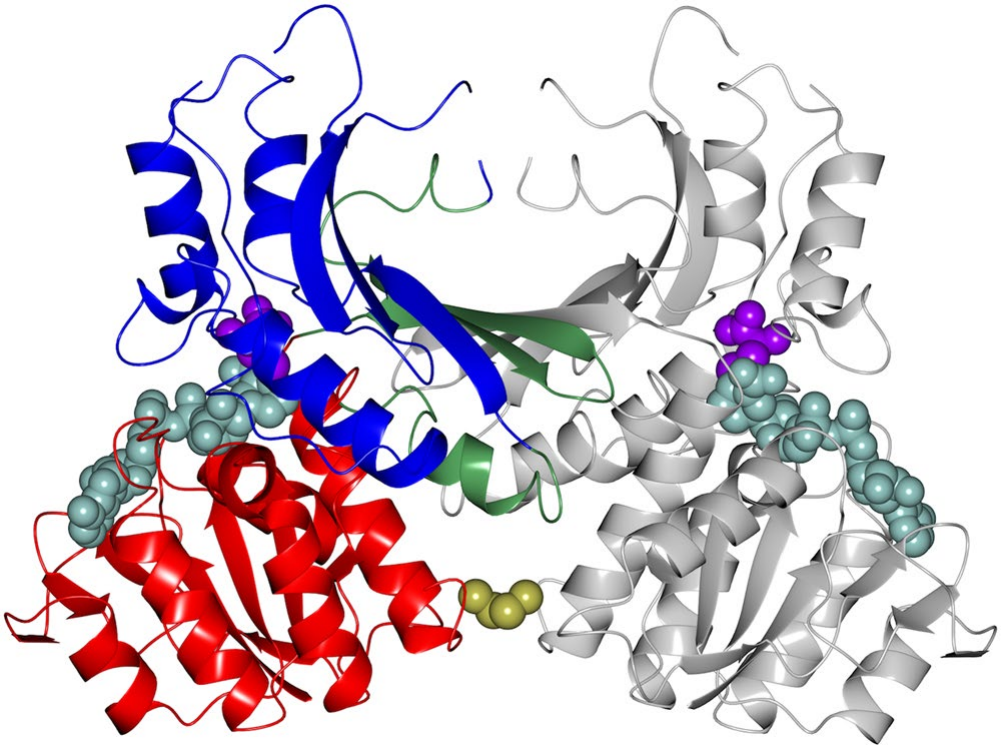
The overall structure of StHSD in its ligand-bound, dimeric form. The nucleotide-binding (residues 1–130 and 285–304), dimerization (residues 131–145 and 256–284), and catalytic (residues 146–255) regions of one monomer are shown in red, green, and blue, respectively. The other monomer is shown in gray. The atoms of the cysteine and NADP are shown as purple and cyan spheres, respectively. The disulfide bond between the two Cys304 residues are shown as yellow spheres.

**Figure 2:**
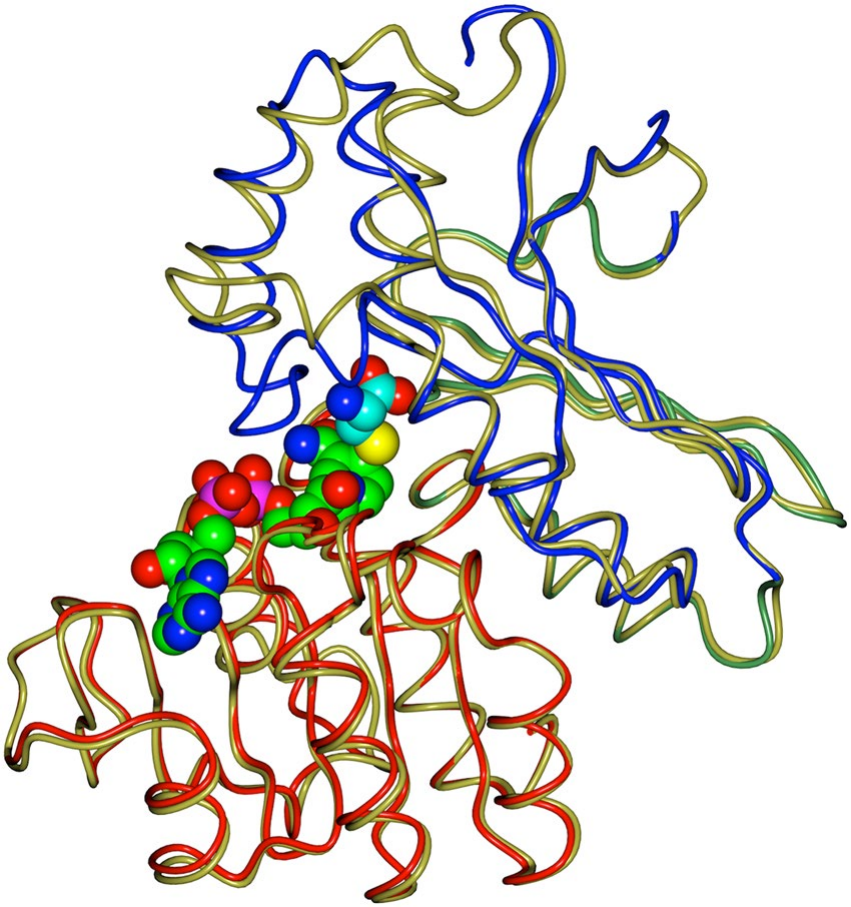
The ligand-bound and native structures of StHSD. The backbone atoms of the ligand-bound structure are superimposed on the native structure. The nucleotide-binding (light red), dimerization (light green), and catalytic (light blue) regions of the ligand-bound form are shown. The atoms of the ligands are shown as spheres with the carbon atoms of cysteine and NAD colored cyan and green, respectively, and with nitrogen, oxygen, phosphorus, and sulfur atoms colored blue, red, pink, and yellow, respectively.

**Figure 3:**
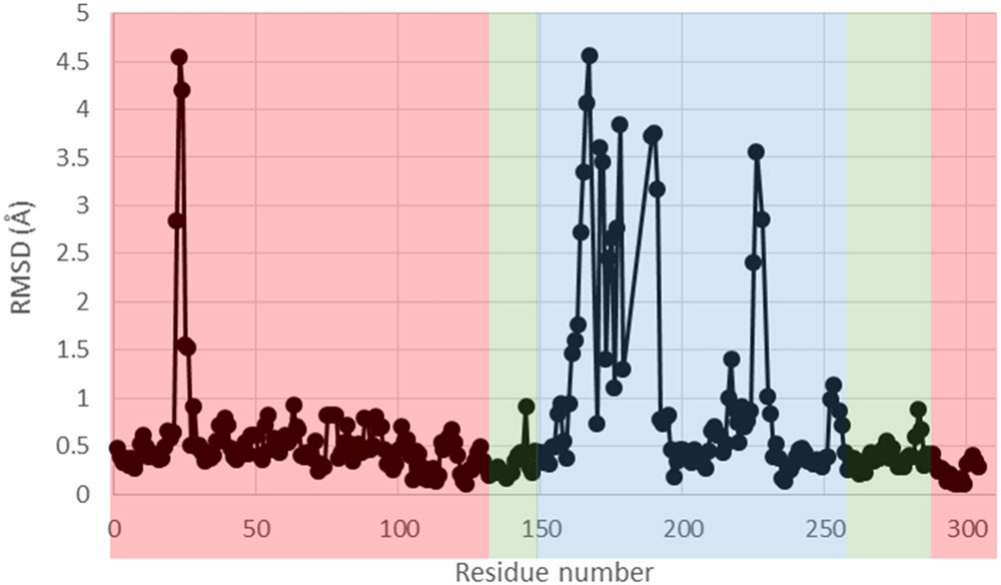
Effects of cysteine and NAD on the backbone atoms. The backbone atoms of the amino acid residues comprising the ligand-bound and native structures are superimposed. The background colors pink, light green, and light blue indicate the nucleotide-binding, dimerization, and catalytic regions, respectively.

Our kinetic analysis showed that StHSD first binds NAD and then homoserine. In addition, cysteine acts as a competitive inhibitor of homoserine but not NAD. This indicates the enzyme reaction is regulated through abortive formation of a cysteine-NAD-enzyme ternary complex. To determine the structural features of cysteine’s inhibition of StHSD, we examined the crystal structure of StHSD in complex with cysteine and NAD at 2.1 Å resolution. The overall structure clearly showed the presence of a disulfide bond formed between two cysteine residues (position 304) in the C-terminal regions of the two subunits (Fig. 1), suggesting that the structure is the less active oxidized form^6^. Superposition of the native and ligand bound structures showed that a large conformational change occurs upon ligand binding, which affects the catalytic region (amino acids 146–255) in particular (Fig. 2). The displacement of backbone atoms by the ligand binding also revealed the conformational change in the catalytic region (Fig. 3). The nucleotide binding regions (amino acids 1–130 and 285–304) had a Rossmann fold characterized by a six-stranded parallel β-sheet. Ligand binding induced much less displacement of the nucleotide binding region than the catalytic region (amino acids 146–255). The conformational change at amino acids 21–26 within the nucleotide binding region (Fig. 3) could be explained by crystal packing contacts, which were found in the structure of the ligand-bound form.

**Figure 4:**
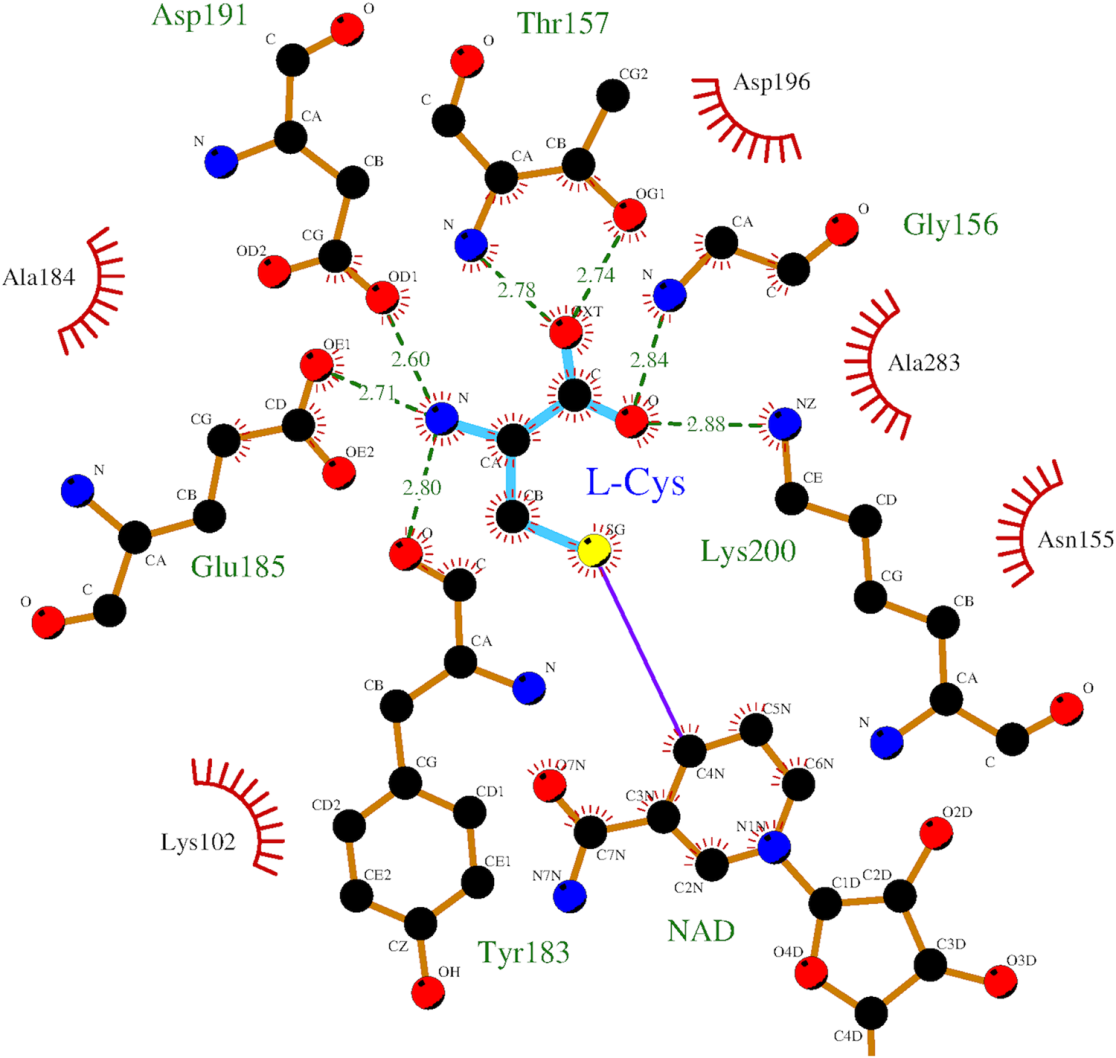
Schematic representation of the cysteine binding residues. Cysteine is shown as balls and cyan bars. Hydrogen bonds are indicated by green dashed lines, and hydrophobic contacts are represented by red arcs with spokes towards cysteine.

**Figure 5:**
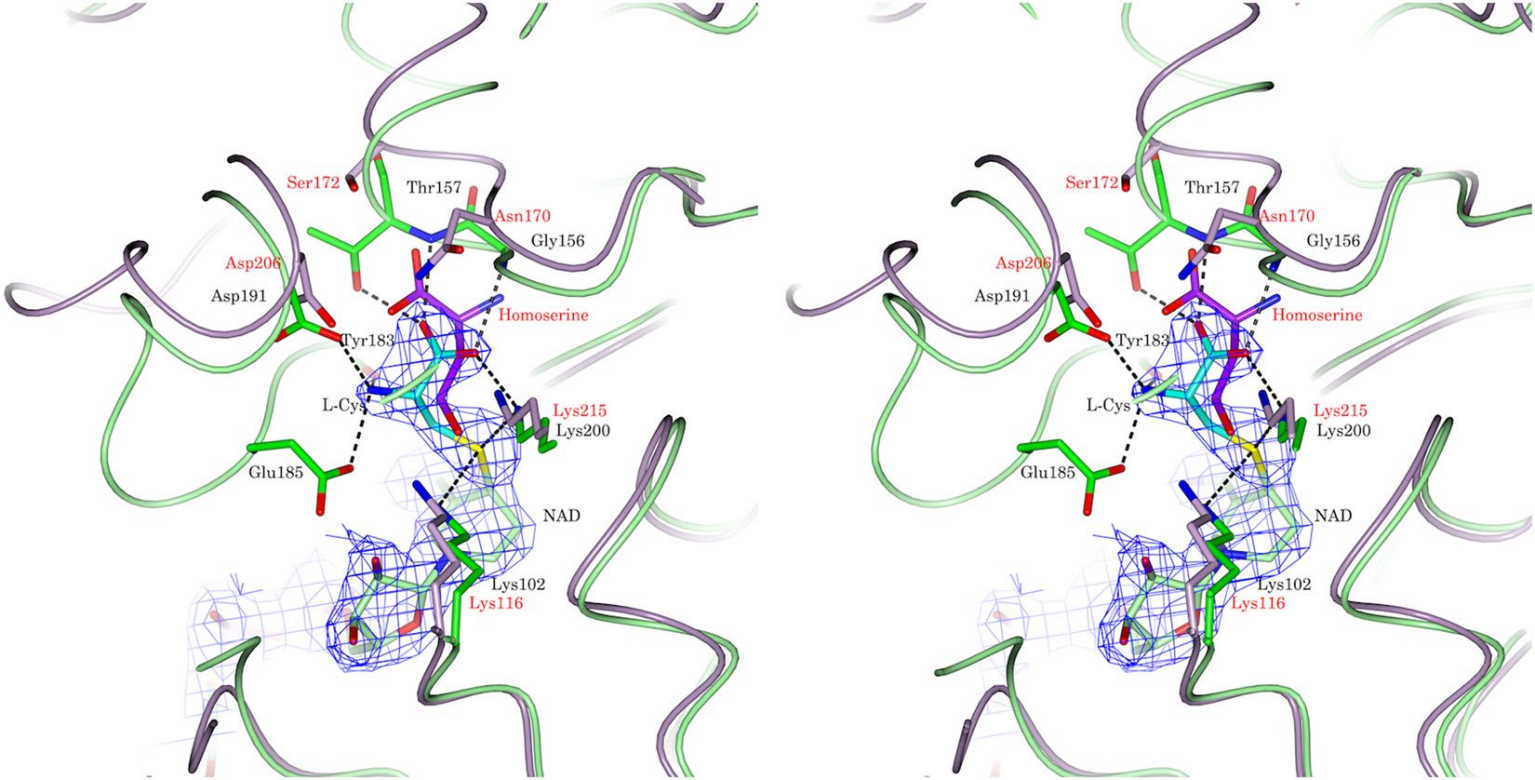
Cysteine binding site. The cysteine (cyan) binding site in StHSD (green and black labels) is superposed on the homoserine (magenta) binding site in PhHSD (light pink and red labels). The *F*o-*F*c omit densities of the substrates are shown in blue at a contour level of +3 sigma levels.

**Figure 6:**
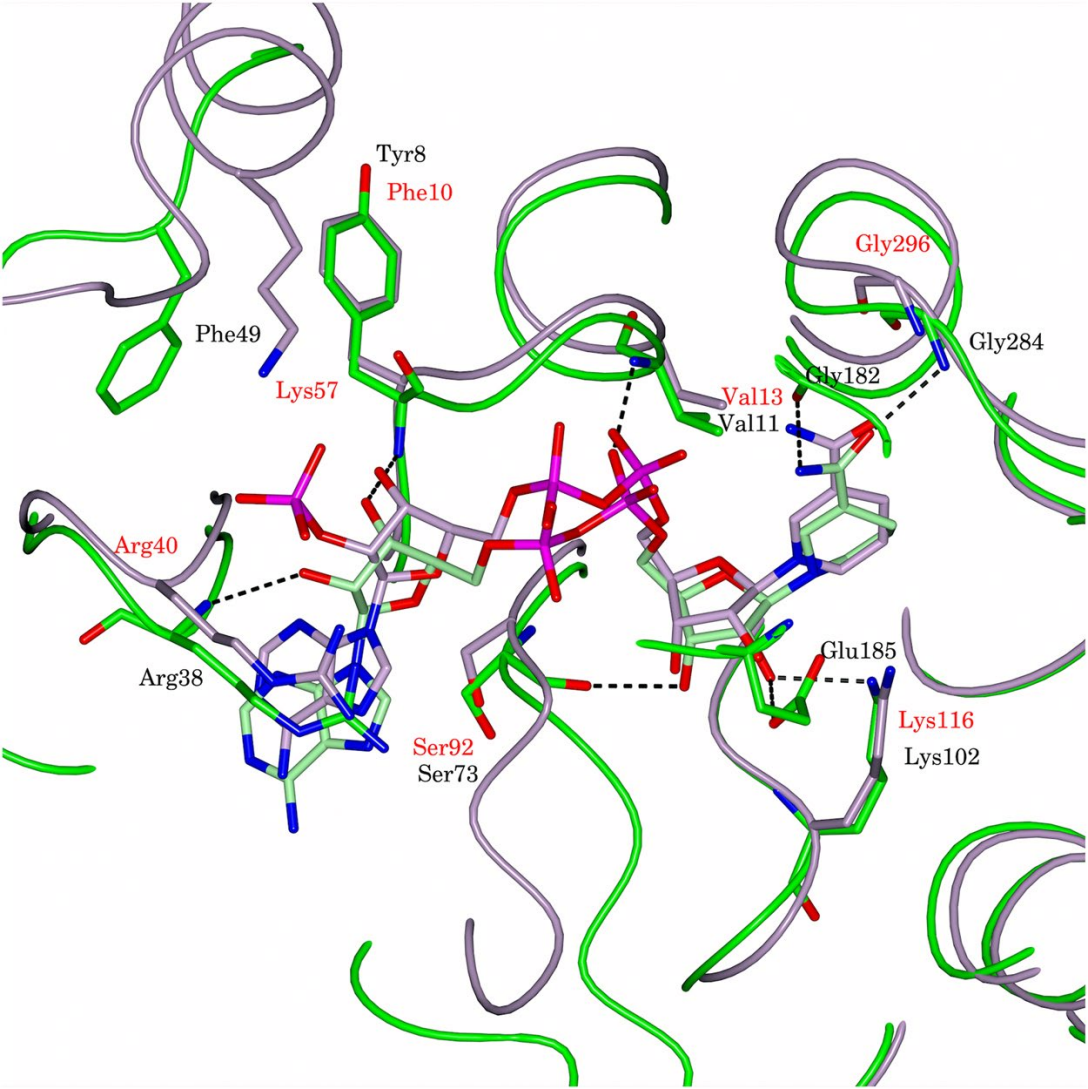
Nucleotide binding site. The NAD binding sites in StHSD (green and black labels) and PhHSD (light pink and red labels) are superposed.

The location and orientation of NAD and cysteine within the active site of StHSD were determined on the basis of the electron density. Cysteine interacted with six residues (Gly156, Thr157, Tyr183, Glu185, Asp191, and Lys200) in the StHSD active site (Fig. 4). When the active site residues of StHSD were superimposed on the corresponding residues of PhHSD harboring homoserine (Fig. 5), it was observed that PhHSD lacks residues corresponding to Tyr183 and Glu185 of StHSD. Interestingly, the distance between the sulfur atom of cysteine and the C4 atom of the nicotinamide ring of NAD was calculated to be 1.9 Å, which is sufficiently close to form a covalent bond. When NAD in the active site of StHSD was superimposed on that of PhHSD in complex with NADP (Fig. 6), the NAD-interacting residues were fully conserved between the two structures, except for Lys57, which tightly interacts with the C2′ phosphate group of NADP. In StHSD, Phe49 is located at the position of the Lys57 residue of PhHSD, and NADP inhibition was not observed. In fact, substituting Lys57 of PhHSD with Ala reportedly makes NADP activity possible^5^.

### Difference UV spectra

**Figure 7:**
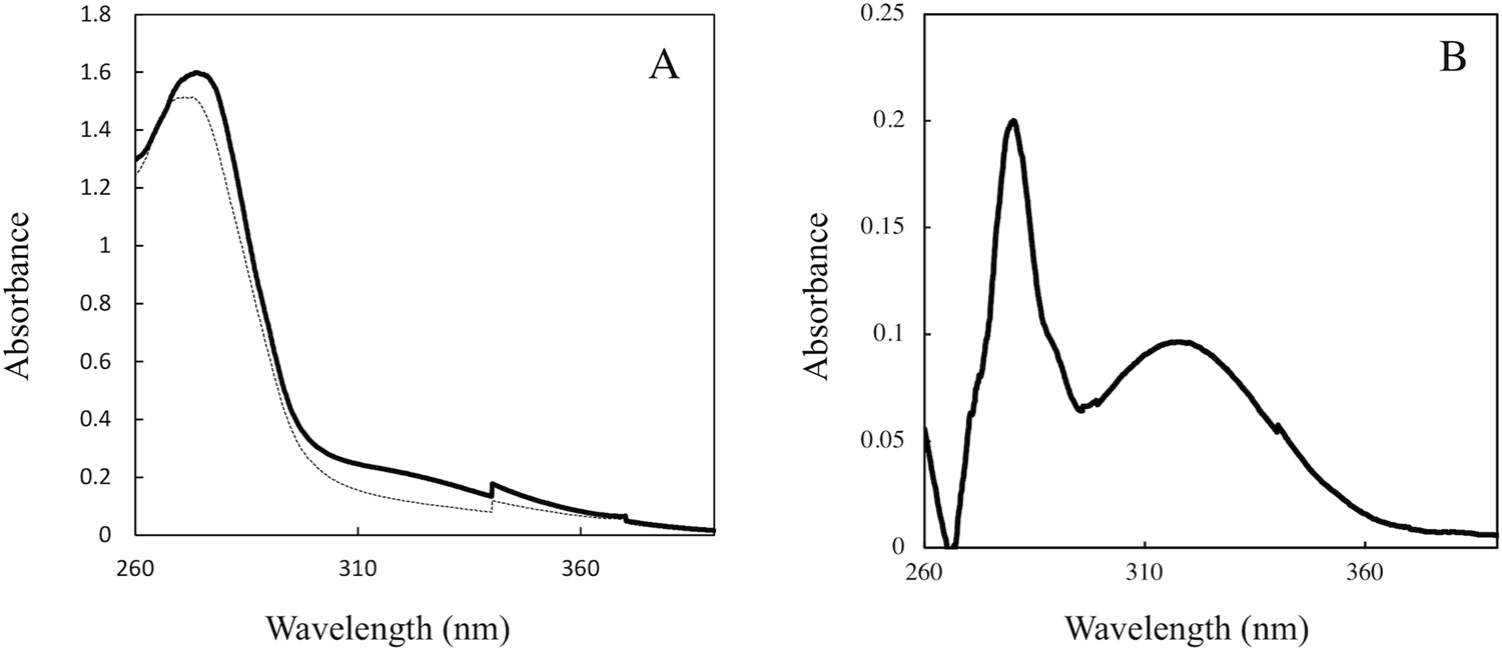
The UV spectra. (**A**) The UV spectra for StHSD plus NAD in the presence (bold line) and absence (thin line) of cysteine. The difference UV spectrum (**B**) was obtained by subtracting the spectra of 20 μM NAD and the enzyme (30 μM) from those of the mixture in the presence of 1 mM cysteine.

Addition of 1 mM cysteine to StHSD in presence of 20 μM NAD induced a new peak at 320 nm in the difference UV spectrum (Fig. 7). This peak suggests the formation of a covalent bond between the C4 atom of the nicotinamide ring of NAD and the sulfur atom of cysteine, as reported^8,9^. Separately, NAD and StHSD showed no peak in this region.

## Discussion

Cysteine markedly inhibited StHSD activity toward homoserine with a low *K*i (11 μM) and may be a physiological inhibitor of StHSD, as its *K*i is around its concentration (15 μM). in *Arabidopsis thaliana* cells^10^ and is consistent with those of other enzymes showing feedback inhibition by cysteine. For example, serine *O*-acetyltransferase isotypes from *Entamoeba histolytica* are inhibited by cysteine with *K*i values ranging from 4.7 to 460 μM^11^. HSD from *E. coli* is inhibited by cysteine, which provides the sulfur for methionine synthesis, and this inhibition is thought to prevent methionine overproduction under conditions in which the cysteine concentration is high^12^. StHSD is thought to be regulated by cysteine for similar prevention of methionine overproduction in *S. tokodaii*. However, within the *S. tokodaii* genome, no orthologous genes required for methionine synthesis from cysteine, such as cystathionine β-lyase (EC 4.4.1.8), have yet been detected.

### Cysteine binding site

Cysteine competitively inhibited StHSD-catalyzed homoserine oxidation. Surprisingly the structural analysis showed that cysteine locates within the homoserine binding site within the StHSD-NAD complex, where it interacts with six amino acid residues (Lys102, Gly156, Thr157, Tyr183, Glu185 and Asp191) (Fig. 5). Superposition of the NADP-homoserine-PhHSD complex with StHSD in complex with NAD and cysteine revealed that the active site residues are conserved in the two HSDs, except for Thr157 and the two residues (Tyr183 and Glu185) of a stretch of the loop (amino acids 165–191). These three residues may function to bind cysteine, rather than contribute to the catalytic activity, and may be responsible for the inhibition of StHSD by cysteine. StHSD possesses amino acid residues that are equivalent to those for the homoserine-binding in PhHSD (the side-chain atoms of Lys116, Asp206 and Lys215 and the backbone N atoms of Ala171 and Ser172)^5^. These residues seem to have function for homoserine binding (Fig. 5). However, Asp191 of the loop in StHSD interacts with the amino group of cysteine, though the equivalent residue Asp206 which interacts with carboxyl group of homoserine in PhHSD^5^. Considering that the significant conformational change of the loop is induced by the cysteine binding (Fig. 3), the displacement of the loop may have important role for cysteine binding.

Both the crystal structure and the difference UV spectra indicate the presence of a covalent bond between the sulfur atom of cysteine and the C4 atom of the nicotinamide ring of NAD, which suggests the inhibitor cysteine molecule binds directly to the NAD coenzyme. We suggest this binding increases the binding affinity of cysteine for the active site. Consistent with that idea, the *K*i for cysteine is much lower than the *K*m for homoserine. In spite of the covalent bond formation, cysteine acted as a competitive, and thus a reversible inhibitor of homoserine. The covalent bond may be transient and readily dissociate by a elimination reaction like the covalent bond between NAD and cysteine residue observed in aldehyde dehydrogenases^8,13^. These enzymes forms the covalent bond between C4 or C2 atom of the nicotinamide ring of NAD and cysteine residue to hinder the efficiency of catalysis. Furthermore, a covalent bond between NAD and substrate during catalysis has been reported. The covalent bond between the C2 atoms of the nicotinamide ring and the substrates is formed during the catalytic cycle of NAD(P)-dependent enzymes^14,15^. To our knowledge, this is the first report showing formation of a covalent bond between NAD and free cysteine at the active site of an enzyme. It may thus be a totally novel mechanism of suppression of the aspartate pathway.

Five residues at the nucleotide binding site of StHSD are conserved in PhHSD. Phe49, however, is substituted by a lysine residue in PhHSD^5^. With PhHSD, NADP does not act as a coenzyme, but as an inhibitor. By contrast, StHSD shows activity toward NADP (apparent *V*max: 0.18 U/mg), though it is much less than toward NAD (apparent *V*max: 1.3 U/mg). Structural comparison of StHSD and PhHSD (Fig. 6) showed that Lys57 of PhHSD tightly interacts with the C2′ phosphate group of the adenine ribose of NADP. A PhHSD K57A mutant reportedly shows greater activity than the wild-type enzyme towards NADP, and the strong binding to NADP may be responsible for the cofactor specificity^5^. Within the structure of StHSD, Lys57 is substituted by Phe49. The bulky and hydrophobic side chain of Phe49 could exclude the C2′ phosphate group of NADP so that Phe49 may be responsible for the low activity toward NADP.

## Methods

### Purification

Recombinant StHSD was overproduced in *Escherichia coli* BL21 (DE3) at 310 K, as described previously^6^. The crude extract prepared in 10 mM Tris-HCl buffer (pH 8.0) was treated for 3 h at 343 K, then centrifuged at 10,000 × *g* for 20 min at 277 K. StHSD in the supernatant was purified through single-column chromatography with DEAE-TOYOPEARL. StHSD was eluted with 10 mM Tris-HCl (pH 8.0) containing 50 mM NaCl after washing with the same buffer without NaCl. The purified enzyme was dialyzed against 10 mM Tris-HCl (pH 8.0) containing 1 mM MgCl_2_ and concentrated using an Amicon Ultra 10 K filter unit (Merck Millipore, Darmstadt, Germany). The homogeneity of the final preparation was confirmed by sodium dodecyl sulfate polyacrylamide gel electrophoresis (SDS-PAGE).

### Enzyme and protein assays

The standard assay was performed at 303 K in a reaction mixture containing 100 mM Tris-HCl buffer (pH 8.0), 10 mM homoserine, 10 mM NAD, and 0.02 mM dithiothreitol. StHSD was reduced prior to use by incubation in the presence of 0.9 mM dithiothreitol for 2 h at 277 K. HSD activity was determined by measuring the rate of NADH formation based on the absorption at 340 nm (molar coefficient: 6,220) at 303 K. The protein concentration was measured using a Pierce BCA protein assay kit (Thermo Scientific, Inc., Waltham MA). One unit of the enzyme was defined as the amount of the enzyme that produced 1 μmol NADH per 1 min at 303 K. The types of inhibition were determined using Lineweaver-Burk plots, and the *K*i values were calculated using Dixon plots.

### Structural determination

Crystals of StHSD were grown in protein solution consisting of 2 μL of purified enzyme (5.0 mg/mL), an equal amount of reservoir solution [23% (w/v) PEG 3350, 0.2 M di-ammonium tartrate], 1 μL of 20 mM NAD^+^, and 1 μL of 100 mM cysteine at 285 K using the hanging-drop vapor diffusion method with 100 μL of reservoir solution. For X-ray diffraction experiments, the crystals were quick-soaked for 15–20 s in reservoir solution containing 20% (v/v) glycerol as a cryoprotectant. Diffraction data were collected at beamline BL-5A at the Photon Factory, Tsukuba, Japan. All data sets were collected at 95 K. The diffraction data were processed using XDS^16^, POINTLESS^17^, and SCALA^18^ in the CCP4 package^19^, after which the datasets were phased using molecular replacement with the program Morlep^20^. The StHSD structure (PDB entry: 4YDR)^8^ was used as an initial phasing model for the structure in complex with cysteine and NAD. The models were built using the program COOT^21^ and refined using Refmac5^22^. NAD, cysteine, and tartrate molecules were identified through examination of the 1*F*o-*F*c and 2*F*o-*F*c electron density maps. The programs RAMPAGE^23^ and SFCHECK^24^ in the CCP4 package were used for stereochemistry analyses of all models and for calculation of the root mean squared deviation and average error using Luzzati plots. The statistics for data collection and refinement are presented in Table 1. All figures illustrating these structures were prepared using CCP4 mg^25^. The coordinates of the structure were deposited in the PDB under entry number 5X9D.

**Table 1:** Data collection and refinement statistics^c^.

Beamline	NE5A (PF)
Wavelength (Å)	1.0000
Resolution (Å)	37.54–2.10
No. of reflections (measured/unique)	139123/18629
*R*_merge_^a^ (%)	8.0 (93.2)
Completeness (%)	100 (100)
Multiplicity (%)	7.5 (7.5)
<*I*/σ(*I*)>	18.4 (2.3)
Overall *B* factor from Wilson plot (Å^2^)	33.06
No. of crystals	1
Space group	*I* 4_1_
Unit –cell constants	
*a* (Å)	106.17
*b* (Å)	106.17
*c* (Å)	56.87
α (°)	90
β (°)	90
γ (°)	90
Refinement statistics	
Resolution range (Å)	50.0–2.10
No. of reflections	18622
*R*_factor_ for 95% data^b^	0.1896
Free *R*_factor_ for 5% data	0.2394
No. of atoms	
Protein	2286
Water	126
Ligand	61
RMSD from ideality	
Bond lengths (Å)	0.0091
Bond angles (°)	1.4520
Average B factors (Å^2^)	
Protein	39.27
Water	43.01
Ligand	30.90
Ramachandran analysis	
Favored (%)	96.6
Allowed (%)	3.4
Disallowed (%)	0

^a^*R*_merge_ = ∑hkl ∑i|*I*_hkl,_ − <*I*_hkl_>|/∑hkl ∑i *I*_hkl,i_, where I = observed intensity and <I> = average intensity for multiple measurements.

^b^*R*_free_ was monitored with 5% of the reflection data excluded from the refinement.

^c^Values in parentheses are statistics for the highest-resolution shell, whose range is 2.21–2.10.

### Difference UV spectra

The difference UV spectra for StHSD in the presence and absence of cysteine were obtained by subtracting the spectrum obtained with 20 μM NAD and the enzyme (30 μM) from that obtained with the mixture in the presence of 1 mM cysteine. The spectrum was monitored in 10 mM Tris-HCl (pH 8.5) at 25 °C.
